# Supplementary material for: Delayed growth of SK-ES-1 Ewing sarcoma tumor xenografts is associated with reduced Trk and IGF1R pathway markers
Source: Oncotarget. 2026 Aug 7;17:354–67. doi: 10.18632/oncotarget.28911 (PMC13637837; doi:10.18632/oncotarget.28911)
Supplement: Supplementary file 1 [file oncotarget-17-28911-s001.pdf]

Delayed growth of SK-ES-1 Ewing sarcoma tumor xenografts is associated with reduced Trk and IGF1R pathway markers

SUPPLEMENTARY MATERIALS

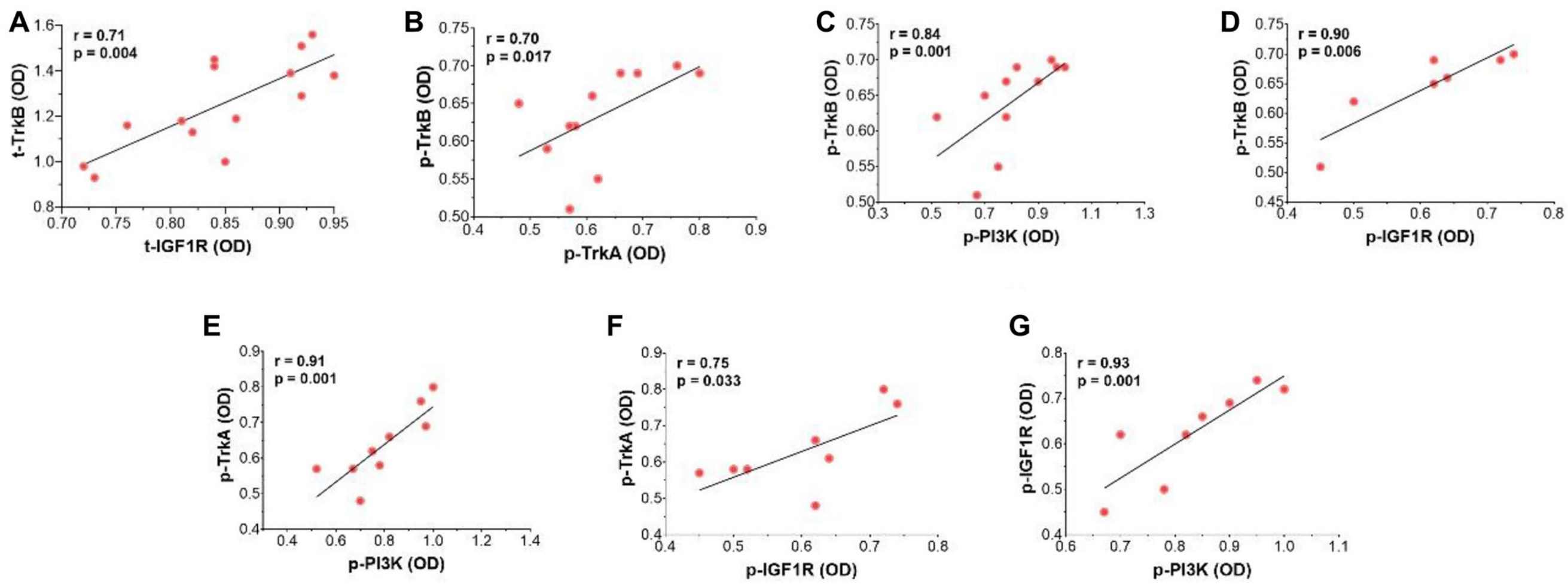

Supplementary Figure 1: Correlation analysis for the expression between (**A**) t-TrkB and t-IGF1R, (**B**) p-TrkB and p-TrkA, (**C**) p-TrkB and p-PI3K, (**D**) p-TrkB and p-IGF1R, (**E**) p-TrkA and p-PI3K, (**F**) p-TrkA and p-IGF1R, and (**G**) p-IGF1R and p-PI3K in ES in ES tumors from both the control and K252a-treated groups, selected on the basis of quality of immunhistochemical staining; *n* = 7–14.

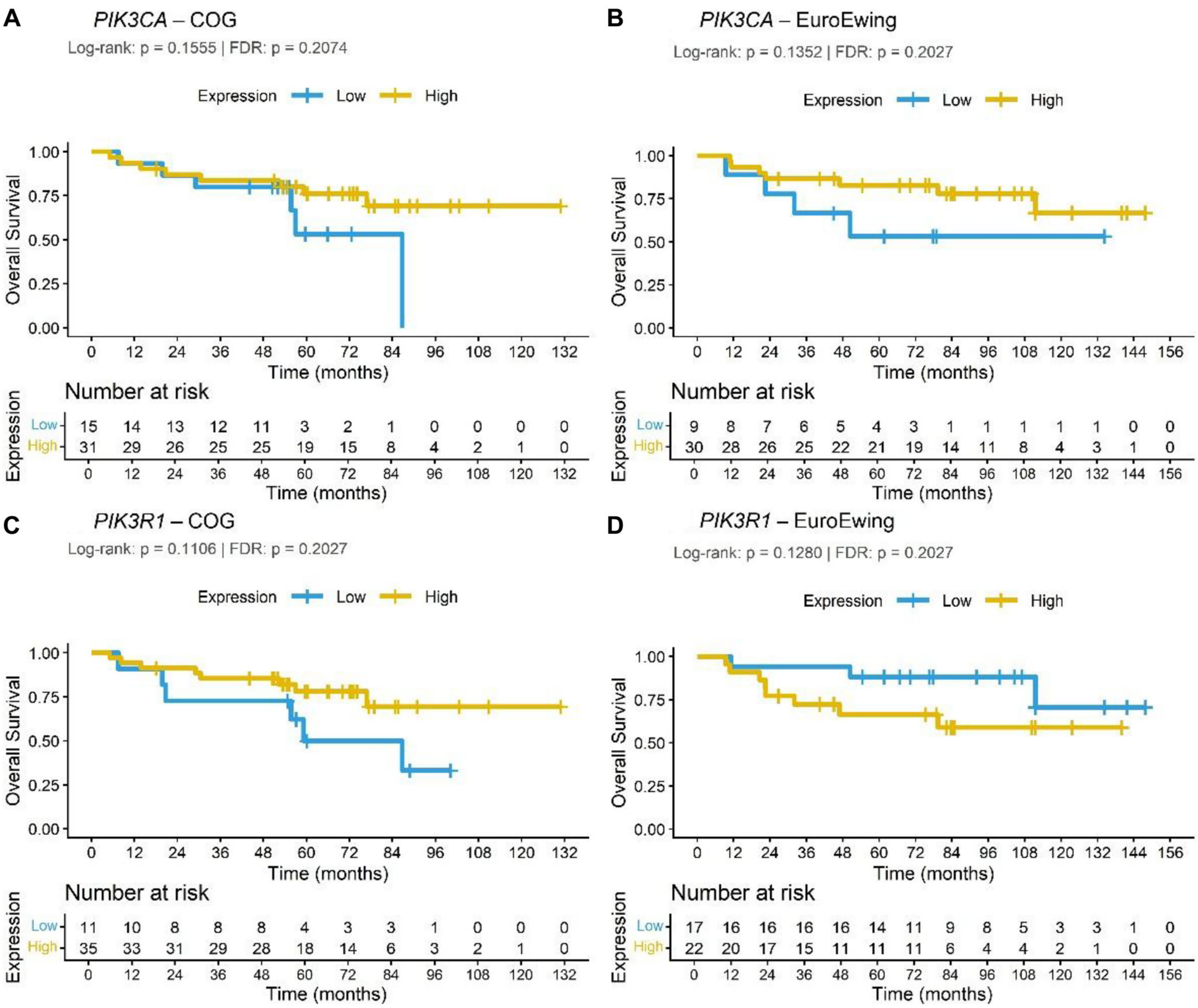

Supplementary Figure 2: Lack of significant associations between expression of genes encoding subunits of PI3K and prognosis assessed by OS in patients with ES. Datasets GSE63155 and GSE63156, comprising expression data from 46 primary ES tumors biopsies from COG and 39 tumors from the EuroEwing collaborative group respectively[28] were used (**A**) *PIK3CA*, COG; (**B**) *PIK3CA*, EuroEwing, (**C**) *PIK3R1*, COG; (**D**) *PIK3R1*, EuroEwing; raw and FDR-corrected *p*-values are indicated in each panel.

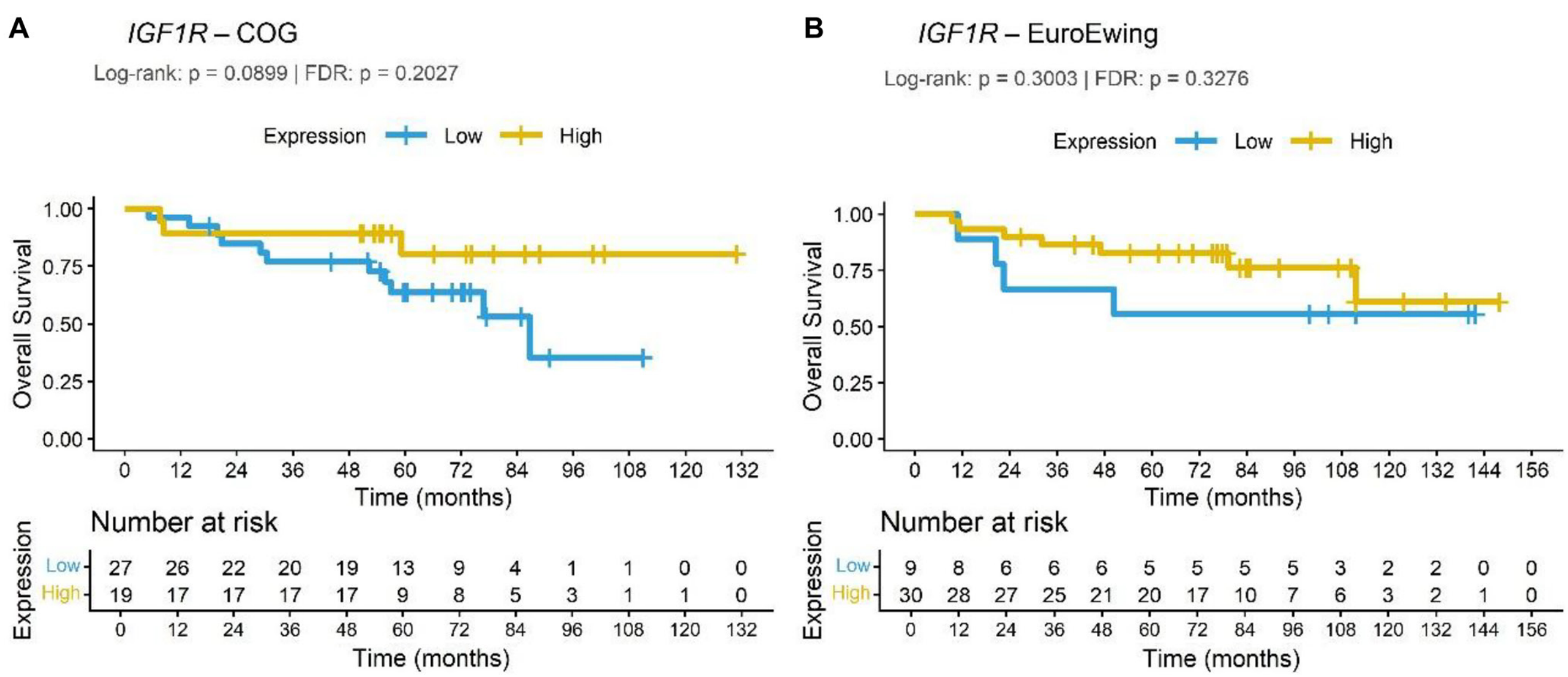

Supplementary Figure 3: Lack of significant association between IGF1R gene expression and prognosis assessed by OS in patients with ES. Datasets GSE63155 and GSE63156, comprising expression data from (**A**) 46 primary ES tumors biopsies from COG and (**B**) 39 tumors from the EuroEwing collaborative group respectively [58] were used; raw and FDR-corrected *p*-values are indicated in each panel.
